# Comparative transcriptome analysis of MDBK cells reveals that BoIFN-γ augmented host immune responses to bovine herpesvirus 1 infection

**DOI:** 10.3389/fmicb.2022.973278

**Published:** 2022-08-09

**Authors:** Bo Jiang, Jing Wang, Wenxiao Liu, Jing Cheng, Jian Xu, Mengyao Cao, Yongqing Li

**Affiliations:** ^1^Institute of Animal Husbandry and Veterinary Medicine, Beijing Academy of Agricultural and Forestry Sciences, Beijing, China; ^2^Lanzhou Veterinary Research Institute, Chinese Academy of Agricultural Sciences, Lanzhou, China; ^3^Animal Science and Technology College, Beijing University of Agriculture, Beijing, China

**Keywords:** bovine herpesvirus 1, interferon-gamma, RNA-Seq, pathogen–host interactions, immune response

## Abstract

Bovine herpesvirus 1 (BoHV-1) is an alphaherpesvirus that causes infectious bovine rhinotracheitis and infectious pustular vulvovaginitis in cattle. Ιnterferon-gamma (IFN-γ) is a pleiotropic cytokine with antiviral activity that modulates the innate and adaptive immune responses. In this study, we prepared high-purity bovine interferon gamma (BoIFN-γ) dimer protein using prokaryotic expression system and affinity chromatography. We subsequently investigated the effect of BoIFN-γ on BoHV-1 infection in Madin-Darby bovine kidney (MDBK) cells. The results showed that BoIFN-γ pre-treament not only decreased the production of BoHV-1 but also reduced the cytopathic effect of the virus. Differential gene expression profiles of BoHV-1 infected MDBK cells were then analysed through high-throughput RNA sequencing. The data showed that BoIFN-γ pre-treatment reduced lipid metabolism disorder and DNA damage caused by BoHV-1 infection. Furthermore, BoIFN-γ treatment upregulated the transcription of interferon regulatory transcription factors (IRF1 and GBP5) and interferon-stimulated genes (ISGs) of MDBK cells. Additionally, BoIFN-γ promotes expression of cellular protein involved in complement activation and coagulation cascades response as well as antigen processing and presentation process, while BoHV-1 infection dramatically downregulates transcription of these immune components including C3, C1r, C1s, PLAT, ITGB2, PROCR, BoLA, CD74, B2M, PA28, BoLA-DRA, and TAPBP. Collectively, our findings revealed that BoIFN-γ pre-treatment can improve host resistance to BoHV-1 infection and regulate transcription or expression of host protein associated with cellular metabolism and innate immune response. This provides insights into the development of prophylactic agents for prevention and control of BoHV-1 infection.

## Introduction

Bovine herpesvirus-1 (BoHV-1), a member of the *Alphaherpesvirinae* subfamily, causes immunosuppression and rhinotracheitis, encephalitis and genital lesions and has led to significant economic loss to the cattle industry worldwide (Muylkens et al., 2007; Rola et al., 2017; Thakur et al., 2017). One key characteristic of BoHV-1 infection is the establishment of latency. Latent viruses are reactivated after cattle are exposed to natural stimuli or corticosteroid treatment. Although vaccination can offer effective protection against the clinical disease, it is difficult to prevent the excretion of recrudescent viruses from latent status (Biswas et al., 2013). BoHV-1 is a successful pathogen because it impairs intrinsic and innate immune responses through encoding genes related to immune-evasion (Jones, 2019). For instance, bICP0 encoded by the IEtu1 promoter plays a role as early interferon antagonist (Saira et al., 2007, 2009). bICP27 is manifested as an inhibitor of IFN-β promoter activation phenomenally (da Silva et al., 2012). Late protein VP8 is a potent IFN antagonist that can disrupt host innate immune responses in the absence of viral protein synthesis (Afroz et al., 2016). gG and UL49.5 would also further antagonise immune-recognition. Since BoHV-1 inhibits immune responses in hosts, it can result in secondary bacterial infection and vaccine breaks (Jones, 2019). Therefore, the severity of disease caused by BoHV-1 is more complicated than its clinical manifestation.

It has previously been demonstrated that the bovine immune response is crucial for the regulation of BoHV-1 latency reactivation (Jones, 2019). BoHV-1 infection stimulates innate and adaptive immune cells to secrete cytokines and antibodies (Decman et al., 2005). One of these is interferon-γ (IFN-γ) which induces or inhibits a number of cellular functions by directly affecting gene expression in many cell types. IFN-γ is important in immune modulation and broad-spectrum pathogen defence, and was originally identified as a secretory factor interfering with viral replication (Kang et al., 2018). Basic components of IFN-γ signalling pathway are defined, activation of IFN-γ signals mainly *via* the Janus kinase (JAK)—signal transducer and activator of transcription (STAT) intracellular signal transduction. Once IFN-γ binds to the receptor, the tyrosine kinases JAK1 and JAK2 phosphorylate the transcription factor (TF) signal transducer and activator of transcription 1 (STAT1), result in nuclear translocation and regulate transcriptions of IFN-γ-inducible genes (Muller et al., 1993; Hu et al., 2021). The activation of the JAK–STAT signalling pathway by IFNs leads to the upregulation of a series of interferon-stimulated genes (ISGs), many of which exert antiviral effect on infected cells (Ezeonwumelu et al., 2021). Increasingly, evidence confirms that IFN-γ acts as a pivotal factor in host defence against double-stranded DNA viruses. Host-derived IFN-γ interferes with viral replication by inhibiting transcription and translation of viral genes (Malmgaard, 2004; Schoenborn and Wilson, 2007). For example, in human papillomavirus (HPV) infection, IFN-γ reduces proteolysis of leucocyte L1 protein, leading to retention of the minor capsid protein L2/genome complex in the late endosome, subsequently inhibiting translocation of the dissociated L2/genome complex into the nucleus for genome replication (Day et al., 2017). Induction of nitric oxide synthase by IFN-γ, and consequent nitric oxide production, was shown to mediate the blocking of vaccinia virus replication after early gene expression (Harris et al., 1995). IFN-γ restricts herpes simplex virus type 1 (HSV-1) reactivation by direct and indirect mechanisms. IFN-γ inhibits HSV-1 mRNA accumulation and the subsequent steps of the viral life cycle including early and late gene expression, viral DNA synthesis and viral replication (Pierce et al., 2005). CD8^+^ T cells producing IFN-γ have been shown to directly suppress immediate early (IE) gene expression and prevent HSV-1 reactivation from latency in neurons (Decman et al., 2005). Accordingly, IFN-γ plays a protective role in acute infection with DNA viruses, especially herpesviruses, but little is known about the role of BoIFN-γ in BoHV-1 infection.

In the present study, we prepared and purified the BoIFN-γ protein in *Escherichia coli* and analysed how pre-treatment with recombinant BoIFN-γ affected BoHV-1 replication in MDBK cells. We further explored the antiviral mechanisms of BoIFN-γ by differential gene expression (DGE) profile to compare gene expression in BoHV-1 infected MDBK cells with and without IFN-γ pre-treatment. The results revealed that BoIFN-γ effectively alleviated MDBK cells from alteration of cellular proteins involved in DNA damage, lipid metabolism disorder and immunocompromise caused by BoHV-1 infection.

## Materials and methods

### High purity BoIFN-γ production

The Bovine IFN-γ (BoIFN-γ) gene encoding 122 amino acids was synthesised by Shenggong Life Technologies (Shanghai) according to the sequence released in GenBank (EU276066, nucleotides 24–390) and ligated into the pET-21a(+) vector (Novagen, Merck KGaA, Darmstadt, Germany) and transformed into Escherichia coli strain BL21 (DE3; Supplementary Table 1). The soluble Bovine IFN-γ protein was prepared as described previously with some modifications (Chen et al., 2010). The BoIFN-γ inclusion bodies were gradually dissolved in the refolding buffer (100 mM Tris–HCl, 2 mM EDTA, 400 mM l-arginine-HCl, 0.5 mM oxidised glutathione and 5 mM reduced glutathione pH 8.0). The soluble BoIFN-γ was purified by gel filtration using a Superdex 200 size exclusion column (GE Healthcare) and anion-exchange chromatography using Resource-Q (GE Healthcare).

### Plaque reduction test of BoIFN-γ

MDBK cell was purchased from the American Type Culture Collection (Manassas, VA, United States). MDBK Cells seeded into 6-well plates at 1 × 10^6^ cells/well were incubated overnight at 37°C, treated with BoIFN-γ in 0.5 μg/well for 12 h and infected with 2 ml BoHV-1 (400TCID_50_/ml) incubated at 37°C and 5% CO_2_ for 1.5 h. An agarose overlay was added to the infected cell monolayer after the virus mixture was removed. The cells were further incubated for 48 h after the layer solidified. When viral plaques became visible, 4% formaldehyde was added to the plate, and viral plaques were counted using 0.1% toluidine blue in saline.

### Cytotoxicity of BoIFN-γ to MDBK cells

MDBK cells were seeded in 6-well plates and cultured to 70% confluency at 37°C for 18–24 h. Then, 1 ml of BoIFN-γ with different concentrations (10, 100, 1,000 U/ml) was added to the culture medium, respectively. Followed by cultivation at 37°C for 12 h, we change the medium containing BoIFN-γ to fresh maintenance medium. Cell proliferation was tested with the CellTiter 96 Aqueous One Solution Cell Proliferation Assay (MTS) Kit (Promega) according to the manufacturer’s instructions, and the OD490 values of the test wells were read with a microplate reader. All tests were performed in triplicate.

### Antiviral activity of BoIFN-γ

The cytopathic inhibition assay was employed to test the antiviral activity of BoIFN-γ on the MDBK cell line. Briefly, Monolayers of MDBK cells were prepared in 96-well plates. The cells were pre-treated with tenfold serial dilutions of the purified BoIFN-γ and incubated 12 h at 37°C 5% CO2 with the maintenance medium (DMEM with 2% foetal bovine serum). The medium containing BoIFN-γ diluent was removed and cells were washed twice with the DMEM medium.100 TCID50 of BoHV-1 was added to cells and absorbed for 1.5 h at 37°C, then the inoculum was removed and cells were washed twice with PBS. And fresh maintenance medium was added to each well and incubated as before (37°C in a CO2 incubator) for 2–3 days. Subsequently, viral CPEs were observed and recorded under an inverted microscope. The unit of antiviral activity was calculated based on the highest dilution that 50% CPE in the cell sheet was inhibited. Each dilution of purified BoIFN-γ was determined in 8 repeats.

### Replication dynamics of BoHV-1 in MDBK pre-treated by BoIFN-γ

After treated with different doses of BoIFN-γ for 12 h, the MDBK cells seeded in 6-well plates were infected with 2 × 105TCID50 of BoHV-1, and viral DNA was isolated from cell and supernatants at different time points. Each experiment was repeated three times. The fluorescence quantitative PCR (FQ-PCR) was performed to calculate DNA copy numbers of BoHV-1in cells using SYBR Green PCR master mix based on gB gene as template (gB PF: 5-GCGAGGAAGAGGAGGAGT-3; gB PR: 5-CATCGGAAGCTGCTGGTAC-3). Viral end-point titration was performed to measure progeny virus titre from supernatants. 50% tissue culture infective dose (TCID50) was determined by Reed–Muench method.

### High-throughput RNA-sequencing

After pre-treatment with 1,000 U/ml BoIFN-γ for 12 h, MDBK cells seeded in 6-well plates were incubated with 2 × 105TCID50 BoHV-1 for 1.5 h and then cultured for 24 h in a maintained medium. Cells from four experimental groups were harvested at 24 h post BoHV-1 infection: Group MIV is infected with BoHV-1 only, group MgIV is pre-treated with BoIFN-γ for 12 h and then infected with BoHV-1, group Mg is pre-treated with BoIFN-γ without BoHV-1 infection and group Mock is control group. Following the instructions of TRIzol Reagent (Life Technologies; Carlsbad, CA, United States), total RNA was extracted from MDBK cells at 24 h post infection of BoHV-1, which were pre-treated with or without BoIFN-γ. The purity and fragment length of RNA was assessed using the NanoDrop spectrophotometer (Peqlab, United States) and Agilent 2,100 Bioanalyzer (Agilent Technologies; Böblingen, Germany). Oligo (dT) magnetic beads were used to enrich RNA. Following the first single strand of cDNA was synthesised, double-stranded cDNAs were purified and selected by AMPure XP beads (Beckman Coulter, Krefeld, Germany) and then construct the cDNA library. The insertion fragment size and the effective concentration were detected and accurately quantified by Agilent 2,100 Q-PCR to achieve the highest quality library standards. RNA-seq was obtained by using the Illumina high-throughput sequencing platform (NovaSeq 6,000) by the Beijing Allwegene Technology Company Limited (Beijing, China). Tophat2 software was utilised to compare the obtained sequence with the genome reference sequence. Fragments per kilobase of exon model per million mapped reads (FPKM) of each gene were analysed on the basis of length of the gene and read count mapped to this gene. Differential expression analysis was accomplished by employing DESeq. Each experiment was repeated 3 times.

### Bioinformatics analysis of differentially expressed gene sets

GO enrichment analysis was performed by using Goseq software (v1.22). KEGG database (Kyoto Encyclopedia of Genes and Genomes)^1^ was applied to study genes and expression information as a whole network, which contains biochemical metabolic pathway and signal transduction pathway. The pathway enrichment analysis of DGEs was analysed by KOBAS software (v2.0) on the KEGG database.^2^

### Relative quantification of differentially expressed genes by quantitative reverse transcriptase PCR

The RNA used for detecting DGEs was further reverse transcribed into cDNA utilising the FastKing RT Kit (with DNase). The target gene expression levels were normalised to the β-actin gene as reference. A detailed list containing primers for target genes and β-actin is shown in Supplementary Table 2. The quantitative PCR (qPCR) was performed with the following conditions utilising the iTaq universal SYBR Green Supermix and CFX96 real-time system (Bio-Rad): initial denaturation was performed at 95°C for 1 min, followed by 40 cycles of denaturation at 95°C for 15 s and annealing/extension at 60°C for 1 min, with endpoint melting-curve analysis.

### Statistical analysis

The data were statistically analysed using GraphPad Prism. The results were expressed as the mean ± SD or the mean ± SEM, as indicated. Statistical significance between experimental groups was assessed by one-way ANOVA with unpaired two-tailed t test or one-way ANOVA, *p*-value significance codes: ***0.001, **0.01, or *0.05.

## Results

### BoIFN-γ inhibits the replication of BoHV-1 in MDBK cells

**Figure 1:**
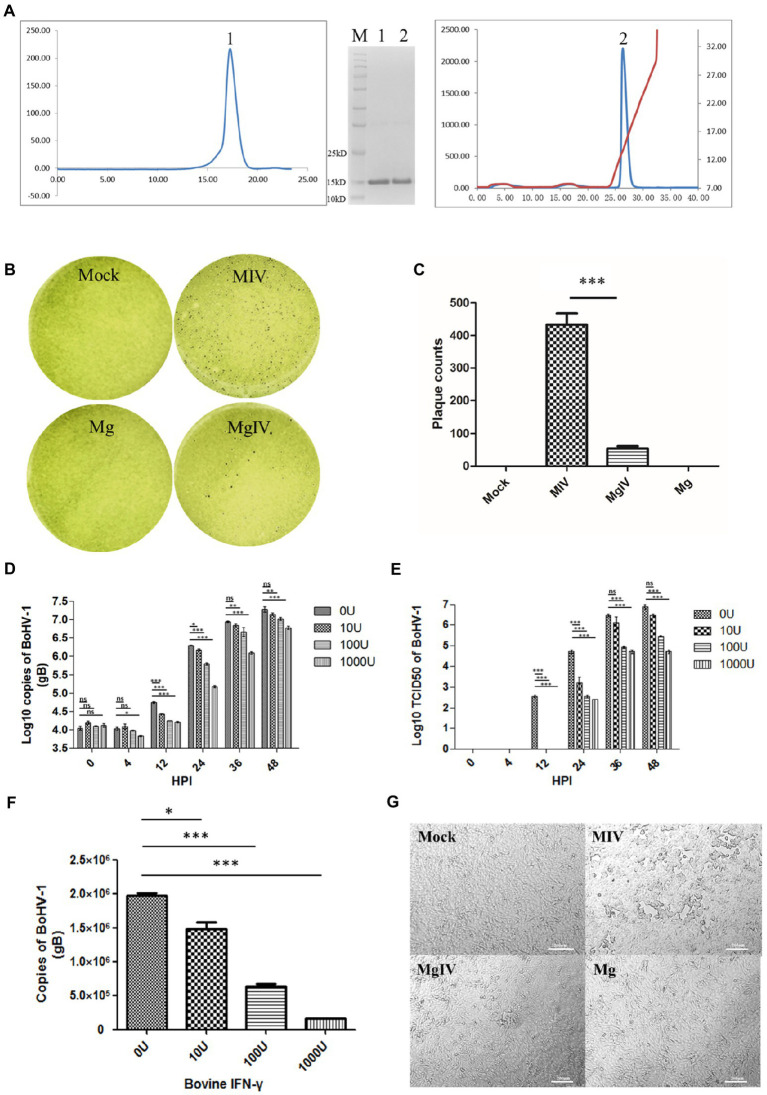
Preparation and antiviral activity of BoIFN-γ. **(A)** The expression and purification of BoIFN-γ, Left, Gel-filtration profile of the BoIFN-γ. Right, Results of further purification of the folded BoIFN-γ by anion-exchange chromatography. The protein was eluted at NaCl concentration of 12.0%–15.0%. Inset, reduced SDS-PAGE gel (15%) of the corresponding purified protein (14.2 kDa). Lane M contains molecular-weight markers (labelled in kDa). **(B,C)** Plaque reduction test of the BoIFN-γ. Mock represents uninfected MDBK cells, MIV represents MDBK cells infected with BoHV-1 and pre-treated with PBS, Mg represents MDBK cells pre-treated with BoIFN-γ only, MgIV represents MDBK cells infected with BoHV-1 and pre-treated with BoIFN-γ. Viral plaques were counted using 0.1% toluidine blue in saline, and reverse the colour to show plaques more clearly. The data were analysed using SPSS software and one-way ANOVA, and the graph was made using GraphPad Prism 5.0. ^***^ represented statistically statistically diﬀerences (*p* < 0.001). ^**^ represents statistically significant differences (*p* < 0.01). ^*^ represents statistically very significant differences (*p* < 0.05). **(D)** Replication curves of BoHV-1 in MDBK cells pre-treated with different doses of BoIFN-γ (10, 100, and 1,000 U/ml) detected use FQ-PCR. **(E)** Replication curves of BoHV-1 in MDBK cells supernatants pre-treated with different doses of IFN-γ (10, 100, and 1,000 U/ml) detected use TCID_50_. **(F)** Replication of BoHV-1 in MDBK cells at 24 h poster infection pre-treated with different doses of BoIFN-γ (10, 100, and 1,000 U/ml) detected use FQ-PCR. **(G)** Changes in cellular morphology at 24 HPI. MIV is MDBK infected with BoHV-1 only, MgIV is MDBK pre-treated 1,000 U/ml BoIFN-γ 12 h and then infected BoHV-1, Mg is MDBK pre-treated BoIFN-γ without BoHV-1 infection, Mock is MDBK cell control.

A total of 6.68 mg of protein was obtained after refolding a 50 mg BoIFN-γ inclusion body. The refolding efficiency of BoIFN-γ inclusion bodies was 10%–13%. Considering the molecular mass of the BoIFN-γ monomer (14.2 kDa), peak 1 (about 28.4 kDa) from size-exclusion chromatography corresponded to a BoIFN-γ dimer (Figure 1A). The protein was then purified by Resource-Q anion-exchange chromatography and the specific peak appeared at a NaCl concentration of 12%–15% (Figure 1A). About 12% SDS-PAGE gel analysis showed a single band corresponding to the expressed BoIFN-γ (14.2 kDa; Figure 1A, inset).

Results of plaque reduction test revealed that BoIFN-γ pre-treatment significantly reduced the number of plaques formed by BoHV-1 in MDBK cells (*p* < 0.01) (Figures 1B,C). The antiviral (BoHV-1) activity of BoIFN-γ in MDBK cells was 4.73 × 105 U/mg (Supplementary Table 3). When compared with the control group treated with phosphate-buffered saline (PBS), MDBK cells treated with three different concentrations of BoIFN-γ (10, 100, and 1,000 U/ml) displayed no obvious cytotoxicity (Supplementary Figure 1).

In order to investigate the replication dynamics of BoHV-1 when inhibited by different doses of BoIFN-γ, MDBK cells seeded in 6-well plates were pre-treated with BoIFN-γ or PBS and infected with 2 × 105TCID50 BoHV-1 for 48 h. Viruses were then isolated from cells and supernatants were obtained at different time points. We subsequently determined DNA copy numbers and titrations. As shown in Figures 1D,E, when compared with the control group, BoHV-1 replication trends were significantly reduced by BoIFN-γ in a dose-dependent manner, especially at 24 h post infection (Figure 1F). These results were verified by examination of the cytopathic effects under light microscopy (Figure 1G).

### Evaluation of transcriptome sequencing data

At least 6.37 GB of clean data were obtained from each sample by transcriptome sequencing. After quality control, the data were further analysed for expression level by using the Illumina NovaSeq 6,000 platform. The Q30 percentages of clean data for all samples were higher than 91.54%, and the GC contents of the clean data for all samples ranged from 46.54% to 58.39% (Table 1). More than 41,496,306 clean reads were obtained from the Mock group, 45,106,246 clean reads from the MIV group, 35,438,468 clean reads from the MgIV group and 47,224, 594 clean reads from the Mg group. At least 92.43% of the clean reads from each panel without BoHV-1 and 63.15% from panels with BoHV-1 were successfully mapped to the bovine reference genome database. In addition, over 90.93% of the clean reads from each panel without BoHV-1 and 61.67% from panels with BoHV-1 were uniquely mapped to the bovine reference genome database. After FPKM standardisation, most of the unique clean reads show only 1 FPKM. Clean reads of more than 60 FPKM were 3–6% of each panel. The construction of DGE libraries is outlined in Table 2.

**Table 1:** Summary statistics for sequence quality control and mapped data of sample.

Sample	Raw reads	Raw bases	Clean reads	Clean bases	Error rate	Q20 (%)	Q30 (%)	GC content	Total mapped	Multiple mapped	Uniquely mapped
Mock1	45,480,366	6.82G	44,378,466	6.66G	0.03%	97.95	93.85	51.30%	43,026,488 (96.95%)	555,090 (1.25%)	42,471,398 (95.7%)
Mock2	43,160,072	6.47G	41,496,306	6.22G	0.03%	97.89	93.64	50.56%	40,053,918 (96.52%)	552,862 (1.33%)	39,501,056 (95.19%)
Mock3	44,746,074	6.71G	42,915,262	6.44G	0.03%	97.80	93.38	46.54%	41,087,942 (95.74%)	608,852 (1.42%)	40,479,090 (94.32%)
MIV1	51,231,662	7.68G	45,106,246	6.77G	0.03%	97.53	93.65	53.06%	32,950,636 (73.05%)	407,372 (0.90%)	32,543,264 (72.15%)
MIV2	61,096,760	9.16G	58,418,608	8.76G	0.03%	96.56	91.82	50.74%	50,157,320 (85.86%)	581,446 (1.00%)	49,575,874 (84.86%)
MIV3	66,870,482	10.03G	59,595,576	8.94G	0.03%	97.41	93.22	51.08%	44,931,266 (75.39%)	537,322 (0.90%)	44,393,944 (74.49%)
Mg1	48,128,268	7.21G	47,224,594	7.08G	0.03%	96.80	91.54	48.18%	43,649,800 (92.43%)	709,602 (1.50%)	42,940,198 (90.93%)
Mg2	56,131,542	8.41G	55,139,968	8.27G	0.03%	97.74	93.41	51.71%	52,950,122 (96.03%)	886,668 (1.61%)	52,063,454 (94.42%)
Mg3	51,216,264	7.68G	50,164,130	7.52G	0.03%	97.76	93.43	51.75%	48,275,374 (96.23%)	787,168 (1.57%)	47,488,206 (94.67%)
MgIV1	48,724,732	7.30G	45,307,390	6.80G	0.03%	96.75	92.00	53.80%	35,637,234 (78.66%)	460,964 (1.02%)	35,176,270 (77.64%)
MgIV2	50,299,172	7.54G	45,154,366	6.77G	0.03%	96.88	92.41	54.28%	34,538,518 (76.49%)	467,382 (1.04%)	34,071,136 (75.45%)
MgIV3	42,525,170	6.37G	35,438,468	5.32G	0.03%	96.80	92.79	58.39%	22,380,332 (63.15%)	526,234 (1.48%)	21,854,098 (61.67%)

**Table 2:** Statistical table of the number of genes in different expression levels (fpkm.stat).

FPKM interval	Mock1	Mock2	Mock3	MIV1	MIV2	MIV3	Mg1	Mg2	Mg3	MgIV1	MgIV2	MgIV3
0~1	15,899 (55.11%)	15,730 (54.52%)	15,816 (54.82%)	15,812 (54.81%)	15,848 (54.93%)	15,808 (54.79%)	15,839 (54.90%)	16,035 (55.58%)	15,910 (55.15%)	15,597 (54.06%)	15,874 (55.02%)	17,479 (60.58%)
1~3	1984 (6.88%)	2024 (7.02%)	2,137 (7.41%)	2,768 (9.59%)	2,790 (9.67%)	2,785 (9.65%)	2,202 (7.63%)	2,195 (7.61%)	2,132 (7.39%)	2,626 (9.10%)	2,550 (8.84%)	2,406 (8.34%)
3~15	5,087 (17.63%)	5,204 (18.04%)	5,154 (17.86%)	5,613 (19.46%)	5,594 (19.39%)	5,655 (19.60%)	5,220 (18.09%)	5,102 (17.68%)	5,073 (17.58%)	5,406 (18.74%)	5,411 (18.75%)	4,749 (16.46%)
15~60	4,354 (15.09%)	4,393 (15.23%)	4,248 (14.72%)	3,744 (12.98%)	3,676 (12.74%)	3,697 (12.81%)	4,042 (14.01%)	3,920 (13.59%)	4,110 (14.25%)	4,096 (14.20%)	3,946 (13.68%)	2,944 (10.20%)
>60	1,527 (5.29%)	1,500 (5.20%)	1,496 (5.19%)	914 (3.17%)	943 (3.27%)	906 (3.14%)	1,548 (5.37%)	1,599 (5.54%)	1,626 (5.64%)	1,126 (3.90%)	1,070 (3.71%)	1,273 (4.41%)

### Identification of differentially expressed genes

**Figure 2:**
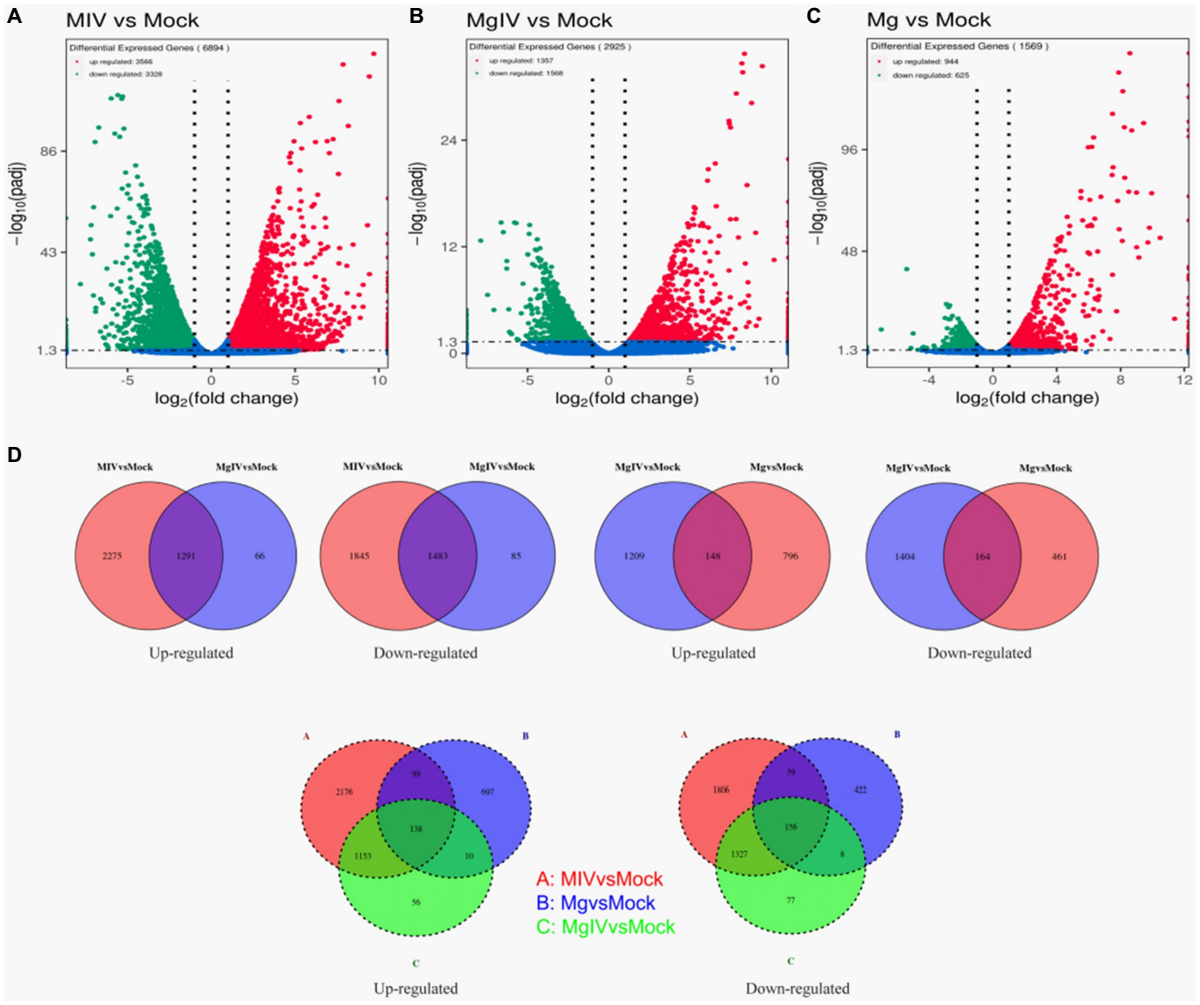
Volcano plot and Venn image of global DEGs in different comparison groups. Volcano plot of global DEGs in different comparison groups. **(A)** MIV vs. Mock. **(B)** MgIV vs. Mock. **(C)** Mg vs. Mock. **(D)** Venn diagrams representing the genes in common among treatment groups. (The statistical criteria are padj < 0.05, |Log2FC| > 1.)

Differentially expressed genes (DEGs) were identified from the different experimental panels (padj < 0.05, |Log2FC| > 1). BoHV-1 infection led to 3,566 upregulated and 3,328 downregulated genes compared to Mock. BoIFN-γ pre-treatment resulted in only 944 upregulated and 625 downregulated genes compared to Mock, and there were 2,925 DEGs in the MgIV to Mock comparison including 1,357 upregulated and 1,386 downregulated genes. When compared with BoHV-1 infection only, BoIFN-γ pre-treated BoHV-1 infection generated nine upregulated genes and only 1 downregulated gene. Meanwhile, there were 1,448 upregulated and only 1,942 downregulated genes detected in the MgIV and Mg comparisons (Table 3). 1,291 upregulated genes were conserved in the BoHV-1 infected cells with and without BoIFN-γ pre-treatment, and 2,275 upregulated genes were specific for BoHV-1 infected, whereas only 66 upregulated genes were specific for the MgIV group. Correspondingly, 1,483 downregulated genes were shared by both samples with and without BoIFN-γ pre-treatment, 1,845 downregulated genes were specific for MIV group and only 85 downregulated genes were specific for MgIV group. Furthermore, 148 upregulated genes and 164 downregulated genes were common to the experimental groups MgIV and Mg. About 1,290 upregulated genes and 1,404 downregulated genes were specific for the experimental group MgIV and 796 upregulated genes and 461 downregulated genes were specific for the Mg group (Figure 2).

**Table 3:** Number of differentially expressed genes of samples.

Criteria	Groups				
	MIV vs. Mock	Mg vs. Mock	MgIV vs. Mock	MgIV vs. MIV	MgIV vs. Mg
padj < 0.05	Up: 4,502 Down: 4,156 Total: 8,658	Up: 1,350 Down: 1,055 Total: 2,405	Up: 1,357 Down: 1,568 Total: 2,925	Up: 9 Down: 1 Total: 10	Up: 1,488 Down: 1,942 Total: 3,430
padj < 0.05 |Log_2_FC| > 1	Up: 3,566 Down: 3,328 Total: 6,894	Up: 944 Down: 625 Total: 1,569	Up: 1,357 Down: 1,568 Total: 2,925	Up: 9 Down: 1 Total: 10	Up: 1,488 Down: 1,942 Total: 3,430
padj < 0.05 |Log_2_FC| > 1.5	Up: 1,248 Down: 1,424 Total: 2,672	Up: 579 Down: 216 Total: 795	Up: 1,248 Down: 1,424 Total: 1,672	Up: 9 Down: 1 Total: 10	Up: 1,318 Down: 1,694 Total: 3,012

### Enrichment analysis of GO terms and KEGG pathway

**Figure 3:**
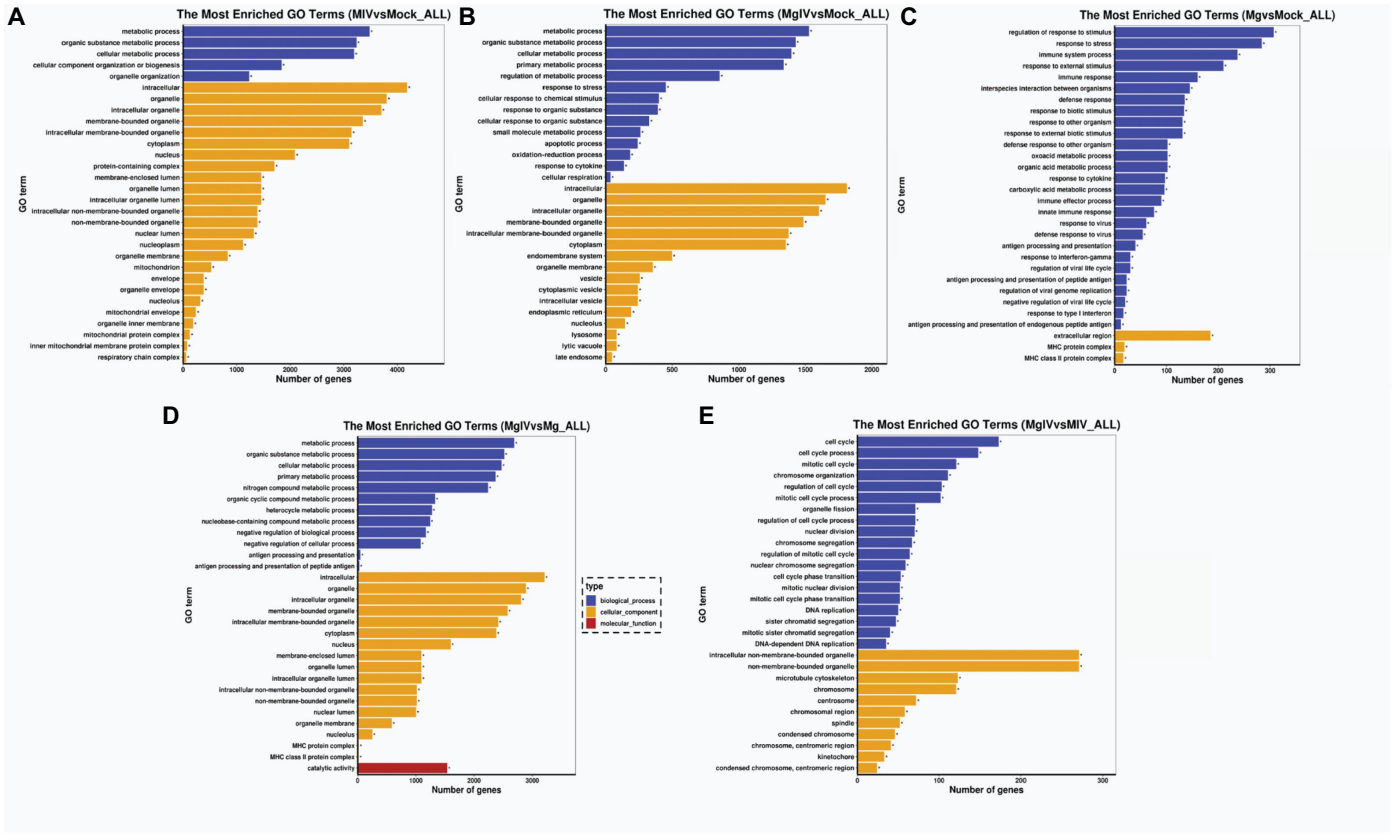
GO enrichment analysis of DEGs in different comparison groups. **(A)** MIV vs. Mock. **(B)** MgIV vs. Mock. **(C)** Mg vs. Mock. **(D)** MgIV vs. Mg. **(E)** MgIV vs. MIV. *p* < 0.05; |Log2FC| > 1. GO terms are on the y-axis. Enrichment ratio of genes shown as GO terms for BP, CC and MF. * means GO categories with significant enrichment.

To determine the functions related to DEGs, we analysed both the GO terms and KEGG pathways. GO classification revealed that the gene expressions altered by BoHV-1 infection were involved in many metabolic processes and cellular components including metabolic process, organic substance metabolic process, cellular metabolic process, organelle organisation, cytoplasm, nucleus, nucleoplasm, mitochondrion, and protein complexes associated with energy supply, such as the inner mitochondrial membrane protein complex and respiratory chain complex and other cellular components-related classifications (Figure 3A). This suggests that viral replication severely affects normal cell metabolism and composition, which is consistent with the cytopathic changes observed. DEGs in the MgIV group significantly enriched not only in metabolic processes and cellular components but also in many of the defence-related processes responsible for maintaining a steady state. These include the regulation of metabolic processes, responses to stress, apoptotic processes, the oxidation–reduction process and responses to cytokine and cellular respiration (Figure 3B). Although BoIFN-γ pre-treatment reduced visible cytopathic changes of MDBK caused by BoHV-1, cell metabolism and cellular component disorders still exist. As expected, regulated DEGs of the Mg group are almost all involved in the host defence-related response, including regulation of the stress response, response to stress, immune system process, response to external stimulus, immune response, innate immune response, interspecies interaction between organisms, extracellular region and major histocompatibility (MHC) protein complex, etc. (Figure 3C). In the comparison between MgIV and Mg groups, not only the metabolism-related processes and cellular components (i.e., metabolic process, organic substance metabolic process, cellular metabolic process, intracellular, organelle and intracellular organelle) were changed, but also many biological processes and cellular components associated with immune responses such as antigen processing and presentation, antigen processing and presentation of peptide antigen, MHC protein complex and MHC class II protein complex. The variety of processes may be seen in Figure 3D. Notably, in the comparison MIV group, MgIV primarily regulated GO terms related to cell cycle-related biology processes such as cell cycle, cell cycle process, mitotic cell cycle and regulation cell cycle process (Figure 3E).

**Figure 4:**
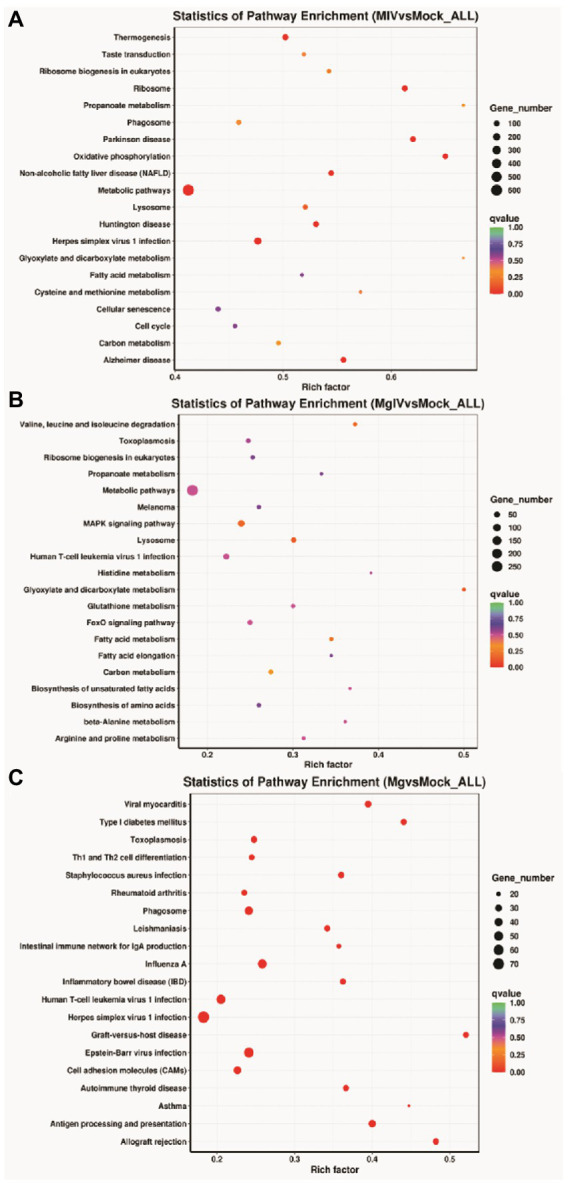
KEGG Pathway enrichment analysis of DEGs in different comparison groups. **(A)** MIV vs. Mock. **(B)** MgIV vs. Mock. **(C)** Mg vs. Mock. The name of KEGG pathways are on the y-axis. q-value (range 0–1) is the *p*-value that was corrected by multiple hypothesis tests. The closer the q-value is to zero, the more significant the enrichment of the KEGG pathway.

The analysis of KEGG pathways revealed that the host response pathways were considerably (*p* value <0.05) affected in all comparison groups 24, 24, 31, 36, and 27 significantly DEG-enriched KEGG pathways were identified in comparison groups MIV vs. Mock, MgIV vs. Mock, Mg vs. Mock, MgIV vs. Mg, and MgIV vs. MIV, respectively, (Supplementary Tables 4–8). The MIV vs. Mock comparison group mainly consisted of pathways focusing on thermogenesis, taste transduction, ribosome biogenesis in eukaryotes, ribosome, propanoate metabolism, phagosome, oxidative phosphorylation, non-alcoholic fatty liver disease (NAFLD), Parkinson disease, metabolism, fatty acid metabolism, carbon metabolism and fatty acid metabolism, lysosomes, eukaryotic ribosome biogenesis, cellular senescence, valine, leucine and isoleucine degradation and antiviral defence pathway including toxoplasmosis, herpes simplex virus 1 and human T-cell leukaemia virus 1 infection (Figure 4A; Supplementary Table 4). The comparison group MgIV vs. Mock pathways primarily included valine, leucine and isoleucine degradation, toxoplasmosis, ribosome biogenesis in eukaryotes, propanoate metabolism metabolic, metabolic pathways, melanoma, fatty acid metabolism, glutathione metabolism, carbon metabolism and some antiviral defence pathways such as the MAPK signalling pathway, FoxO signalling pathway and human T-cell leukaemia virus 1 infection (Figure 4B; Supplementary Table 5). The Mg vs. Mock comparison group primarily included pathways associated with viral myocarditis, type I diabetes mellitus, toxoplasmosis, Th1 and Th2 cell differentiation, staphylococcus aureus infection, rheumatoid arthritis, phagosome, leishmaniasis, antigen processing and presentation, allograft rejection, inflammatory bowel disease, herpes simplex virus 1 infection, human T-cell leukaemia virus 1 infection and toxoplasmosis complement and coagulation cascades, the p53 and NOD-like receptor signalling pathways (Figure 4C; Supplementary Table 6). The MgIV vs. MIV comparison group pathways primarily included staphylococcus aureus infection, leishmaniasis, phagosome, asthma, antigen processing and presentation, Th1 and Th2 cell differentiation, herpes simplex virus 1 infection, cell adhesion molecules and complement and coagulation cascades (Supplementary Table 7). The MgIV vs. Mg comparison group pathways included antigen processing and presentation, graft-versus-host disease, lysosome, valine, leucine and isoleucine degradation, phagosome, metabolic pathways, Epstein–Barr virus infection, type I diabetes mellitus and some immune-related pathways such as Th1 and Th2 cell differentiation, Th17 cell differentiation, human T-cell leukaemia virus 1 infection and p53 signalling pathway (Supplementary Table 8). The KEGG pathways that were significantly enriched in both BoHV-1 infection groups (MgIV and MIV) included metabolic pathways, lysosomes, eukaryotic ribosome biogenesis, carbon metabolism, fatty acid metabolism, valine, leucine and isoleucine degradation and human T-cell leukaemia virus 1 infection. In the BoIFN-γ pre-treated groups (MgIV and Mg), the significantly enriched KEGG pathways included glutathione metabolism, toxoplasmosis and human T-cell leukaemia virus 1 infection.

### Differential expression of representative functional genes

**Figure 5:**
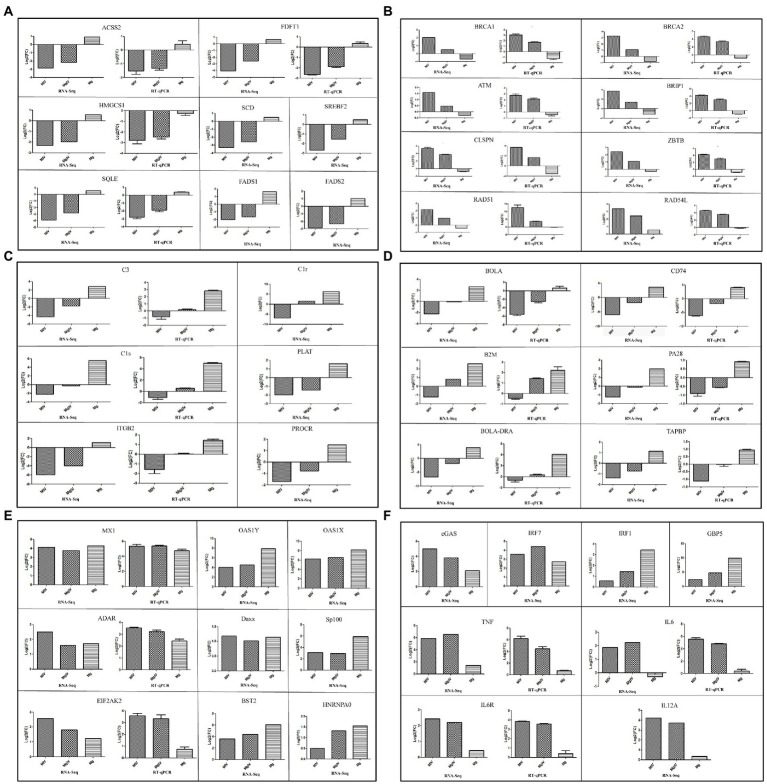
Expression level of genes related to lipid metabolism, DNA damage and repair, interferon stimulation genes, complement and coagulation signalling cascades, antigen processing and presentation, interferon regulatory factors and pro-inflammatory cytokines. ACSS2, FDFT1, HMGCS1, SQLE, BACA1, BACA2, ATM, BRIP1, CLSPN, ZBTB, RAD51, RAD54L, C3, C1S, ITGB2, BoLA, CD74, B2M, PA28, BoLA-DRA, TAPBP, MX1, ADAR, EIF2AK2, TNF, IL6, and IL6R validated by RT-qPCR. **(A)** Expression level of genes related to lipid metabolism. **(B)** Expression level of genes related to DNA damage. **(C)** Expression level of genes related to complement and coagulation signalling cascade. **(D)** Expression level of antigen processing and presentation. **(E)** Expression level of genes related to interferon stimulated genes. **(F)** Expression level of genes related to some interferon regulatory factors and pro-inflammatory cytokines. β-actin gene was used as an internal control and relative quantity of gene expression (fold change) of each gene was calculated with the comparative 2^−ΔΔCT^ method. Values (RT-qPCR) shown were mean with SD.

According to Go and KEGG analysis results, the metabolic response of host cells was significantly disturbed during BoHV-1 infection. Combined with differences in gene transcription levels, we found some DEGs associated with lipid synthesis and metabolism, including ACSS2, FDFT1, HMGCS1, SERBF2, SQLE, FADS1, and FADS2, were significantly downregulated in both comparison group MIV vs. Mock and comparison group MgIV vs. Mock, but transcriptional inhibition of these genes was less in the MgIV group. The results showed that the lipid metabolism disorder caused by virus infection could be improved by BoIFN-γ pre-treatment and the remission of cell cytopathic effects (Figure 5A). Additionally, the genes (BRCA1, BRCA2, ATM, BRIP1, CLSPN, ZBTB1, RAD51, and RAD54L) involved in DNA damage were upregulated in the MIV group, indicating that this occurred as a result of BoHV-1 infection. However, upregulation of these genes in the MgIV group was less with BoIFN-γ pre-treatment, possibly due to inhibition of viral DNA replication by BoIFN-γ (Figure 5B). Compared with the Mock group, BoHV-1 infection (MIV) drastically reduced the transcriptional levels of the gene coding for complement components C3, C1r, C1s, PLAT (TPA), ITGB2 (CD18), and PROCR (EPCR), but gene transcriptional was significantly induced by BoIFN-γ treatment (Mg). Simultaneously, BoIFN-γ pre-treated cells (MgIV) demonstrated significant resistance to negative regulation of these complement-associated genes by BoHV-1 infection (Figure 5C). Furthermore, expression of the BoLA, CD74, B2M, PA28, BoLA-DRA, and TAPBP genes involved in the antigen presentation process of the acquired immune response was suppressed by BoHV-1 infection but also improved by BoIFN-γ pre-treatment (Figure 5D). As expected, transcription of interferon stimulated genes (ISGs), including MX1, OAS1Y, OAS1X, ADAR, BST2, HNRNPA0, and EIF2AK2, was increased by both BoIFN-γ treatment and BoHV-1 infection. Daxx and Sp100, important components of PML-nuclear bodies, were also upregulated both directly by BoIFN-γand indirectly by viral infection (Figure 5E). Furthermore, both cGAS and IRF7 were upregulated in MIV and MgIV groups, suggesting that the type 1 interferon pathway was activated due to the transcription of cGAS and IRF7 triggered in response to viral infection. Meanwhile, the NF-κB pathway was activated by BoHV-1 infection and the transcription of pro-inflammatory cytokines IL6, IL12, and TNF-α was upregulated in the MIV group (Figure 5F).

### Quantitative real-time PCR analysis

Comparisons of transcriptome sequencing data revealed that BoHV-1 infection caused cell metabolism disorder, DNA damage and immunosuppression, while BoIFN-γ pre-treatment enhanced expression of the genes responsible for immune defence and partially inhibited the gene expression involved in apoptosis. To validate the DEGs from our transcriptome sequencing, real-time PCR (RT-qPCR) was used to quantitatively measure the mRNA transcription of 34 selected genes related to lipid metabolism, DNA damage and repair, complement and coagulation signalling cascade responses, antigen processing and presentation, interferon stimulation genes, some cytokines and chemokines and some other immune-related genes, including ACSS2, FDFT1, HMGCS1, SQLE, BACA1, BACA2, ATM, BRIP1, CLSPN, ZBTB, RAD51, RAD54L, C3, C1S, ITGB2, BoLA, CD74, B2M, PA28, BoLA-DRA, TAPBP, MX1, ADAR, EIF2AK2, TNF, IL6, IL6R, ERN1, BCL2L11, GADD34, MAPK8, MAP3K5, ATF4, and CXCL3 (Figure 5; Supplementary Figure 2).

**Figure 6:**
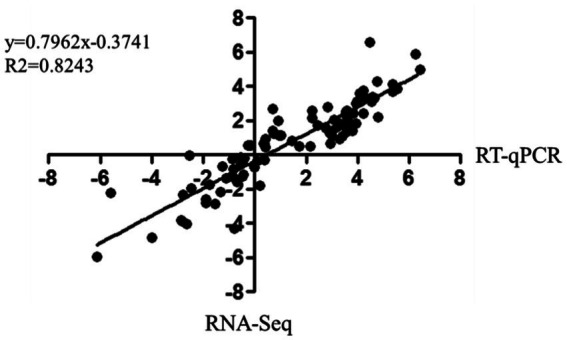
Correlation of fold change analysed by data obtained using RT-qPCR (x-axis) with RNA-Seq platform (y-axis). Correlation analysis was performed using GraphPad software 6.0 (San Diego, CA).

The correlation between RNA-Seq and RT-qPCR results was measured by a scatter plot of the log2 fold-changes. RNA-Seq results were highly correlated with RT-qPCR data, with a correlation coefficient (R2) as high as 0.8243 (Figure 6). In summary, RT-qPCR analysis validated the transcriptional changes of the DEG data from RNA-Seq.

## Discussion

BoHV-1 is a tactical pathogen with various strategies to impair innate and adaptive immune responses throughout productive infection (Jones, 2019). IFN-γ, a pleiotropic cytokine that modulates both innate and adaptive immune networks, was first discovered as a soluble macromolecule with antiviral activity. IFN-γ not only indirectly inhibits virus replication by inducing type I IFNs but also displays type I IFN-independent antiviral activity (Kropp et al., 2011; Hwang et al., 2012; Prestwood et al., 2012). As MDBK cell lines have been widely used to investigate interactions between the host and BoHV-1 and to evaluate novel antiviral approaches, a BoHV-1/MDBK system was employed in this study to investigate the anti-BoHV-1 activity of BoIFN-γ (Wang et al., 2021). The results showed that purified BoIFN-γ significantly reduced the cytopathic effect of productive BoHV-1 infection (Figures 1B,C,G). Furthermore, we evaluated the replication kinetics of BoHV-1 in MDBK cells pre-treated with BoIFN-γ and found that BoIFN-γ halted the replication speed of BoHV-1 in a dose-dependent manner (Figures 1D–F). In order to determine the molecular mechanisms by which BoIFN-γ helps establish an antiviral state in the host, we analysed the differences in gene expression between groups of MDBK cells by RNA-Seq. Transcriptome analysis of MDBK cells revealed that BoHV-1 infection destroyed many biological processes leading to cell death, such as lipid metabolic processes and DNA damage processes. Furthermore, BoHV-1 infection suppressed the transcriptions of these immune components related to complement activation and coagulation cascades response as well as antigen processing and presentation process. However, BoIFN-γ pre-treated cells (MgIV) displayed clearly alleviative injury caused by BoHV-1 infection, which suggests that BoIFN-γ pre-treatment strengthened the resistance of MDBK to BoHV-1 infection.

Replication and infectivity of viruses rely on obtaining all energy and raw materials from the host cell (Cvirkaite-Krupovic et al., 2015). Herpesviruses such as HCMV and HSV-1 actively hijack the metabolic pathways of host cells to create suitable intracellular microenvironments for their life cycle (Vastag et al., 2011; Strating and van Kuppeveld, 2017). In this study, results of enrichment analysis of GO terms and KEGG pathway demonstrated that BoHV-1 infection led to severe metabolic dysfunction. Combined with DEGs significant difference and pathway enrichment analysis, we found that expressions of the genes ACSS2, FDFT1, HMGCS1, SERBF2, SQLE, FADS1, and FADS2, all related to lipid metabolism, were significantly downregulated during BoHV-1 infection. However, these genes were downregulated to a lesser degree by viral infection in BoIFN-γ pre-treated cells (Figure 5F). HMGCS1 is a key ketogenesis enzyme for regulating sterol biosynthesis and cholesterol metabolism and, together with ACSS2, plays an important role in cell growth and progression and cattle growth performance (Wang et al., 2016; Xu et al., 2018). SQLE and SCD are considered rate-limiting enzymes in lipid metabolism that catalyse the synthesis of monounsaturated fatty acids (MUFAs) and inhibitors of the cell death process of ferroptosis (Trapani et al., 2012; Yuan et al., 2020). FDFT1 synthesises squalene *via* condensation of two molecules of farnesyl pyrophosphate, and is critical for metabolic reprogramming, cell proliferation and virus propagation (Park et al., 2014; Ha and Lee, 2020). SREBP2 maintains lipid homeostasis by regulating cholesterol and fatty acid metabolism (Song et al., 2017). Thus, dramatic downregulation of these gene transcriptions indicates that host lipid metabolism was disturbed by BoHV-1 infection, making the normal cell cycle difficult to maintain. Nevertheless, such injuries can be significantly lessened by BoIFN-γ pre-treatment.

As part of the host’s immune surveillance, viral replication is recognised by infected cells as DNA damage leading to apoptosis. In the process of DNA virus replication, exogenous viral DNA structures trigger DNA damage and repair responses in cells (Afroz et al., 2018). In our study, DEGs involved in DNA damage and repair, including BRCA1, BRCA2, BRIP1, ZBTB1, RAD51, RAD54L, ATM, and CLSPN, were significantly upregulated by BoHV-1 infection, but regulation of these genes was relatively improved in group MgIV (Figure 5D). BRCA1 and BRCA2 are required for maintaining chromosomal stability to protect the genome from damage and to transcriptionally regulate some of the genes involved in DNA repair (Yoshida and Miki, 2004). BRIP1 interacts with numerous proteins associated with the regulation of DNA damage responses and signal checkpoints which are vital for retaining chromosomal and genomic constancy (Khan and Khan, 2021). ZBTB1 is a critical upstream regulator of translesion DNA synthesis and promotes chromatin remodelling and translesion DNA for DNA repair (Kim et al., 2014; Zhang et al., 2020). RAD51 and RAD54 are key elements in homologous recombination which is a versatile DNA damage repair pathway. The strand exchange protein RAD51 promotes genome stability by repairing DNA double strand breaks and damaged replication forks. RAD54L acts with RAD51 to promote recombinational DNA repair (Mason et al., 2015; Mun et al., 2020). Upregulation of RAD51 mRNA expression inhibits the STING-mediated innate immune response (Bhattacharya et al., 2017). As a primary regulator in the DNA damage response, ATM activates many signal pathways associated with cell cycle checkpoints, DNA damage repair, transcription regulation, immune response and metabolism (Jackson and Bartek, 2009; Afroz et al., 2018; Xiao et al., 2019). Additionally, ATM has a positive role in V(D)J-recombination through end-tethering and end-processing, ensuring proper end-joining. There is some evidence that ATM promotes survival in developing lymphocytes undergoing V(D)J-recombination (Weitering et al., 2021). Our results demonstrated that BoHV-1 infection upregulated ATM expression leading to activation of the acquired immune response by V(D)J-recombination. Thus, upregulation of gene transcription in the MIV group is sufficient to indicate that BoHV-1 infection can induce DNA damage and further upregulates programmed cell death. The transcriptional levels of these genes in the MgIV group were more like those of the Mock group than those of the MIV group, suggesting that BoIFN-γ pre-treatment not only inhibited viral replication but also alleviated cellular DNA damage. The mechanism by which BoIFN-γ ameliorates DNA damage caused by BoHV-1 infection remains to be further studied.

C1r, C1s and C1q together form C1, the complex which triggers the classical complement pathway. The downregulated expression of C1 and C3 can inhibit activation of the complement system (Flyvbjerg, 2017). C3, C1r, C1s, PLAT (TPA), ITGB2 (CD18), and PROCR (EPCR), which are enriched in complement and coagulation cascade pathways, were downregulated during BoHV-1 infection but exhibited normal levels with BoIFN-γ pre-treatment (Figure 5C). These findings showed that BoHV-1 inhibits the complement system, facilitating replication during the early stages of infection. The same result, that the expression levels of genes belonging to the complement and coagulation signalling cascades were downregulated during BVDV infection, has been previously reported (Liu et al., 2019). However, this inhibitory effect was greatly reduced in our study *via* BoIFN-γ pre-treatment. This suggests that BoIFN-γ can activate innate immunity by inducing the complement system to inhibit virus replication in the early stages of BoHV-1 infection.

Additionally, DEG analyses revealed that BoHV-1 infection significantly inhibited transcription of genes in the antigen processing and presentation pathway including BoLA, BoLA-DRA, TAPBP, CD74, B2M, and PA28. However, this negative regulation caused by BoHV-1 infection is very mild in MDBK pre-treated with BoIFN-γ. It has been reported that BoHV-1 can inhibit antigen processing and presentation in many ways. Koppers’ research show that BoHV-1 interferes with TAP-dependent peptide transport and intracellular trafficking of MHC class I molecules to inhibit CD8+ T cell responses in human cells (Koppers-Lalic et al., 2003; Jones, 2009). BoHV-1 glycoprotein B can also affect CD4+ cell activation by retarding HLA-DR export to the plasma membrane (Grabowska et al., 2020). Previous study reported that IFN-γ could enhance the cytotoxicity of peripheral blood mononuclear leukocytes from immune cattle to BoHV-1-infected cells *in vitro* cytotoxic activity, which is in concert with our transcriptional data that BoIFN-γ initiated transcription of genes in the antigen processing and presentation pathway to CD8+ T cells through MHC I molecule (Campos et al., 1989). Moreover, similar to our results, Fu’s work indicated that transcription of MHC I and II molecules was stimulated by IFN-γ in PK15, suggesting that IFN-γ may strengthen CD4, CD8 and NK cell responses *in vivo* (Fu et al., 2016). This is the first report of BoHV-1 suppressing antigen processing and presentation by inhibiting transcription of related genes in MDBK, although further experiments are required to confirm the effect *in vivo*.

ISGs products, including OAS1, OAS2, MX1, ADAR, and EIF2AK2, are major players in innate immune defence against viral infection. Their expression was upregulated by both BoHV-1 infection and BoIFN-γ pre-treatment in the present study (Figure 5B), indicating that BoIFN-γ provoked the transcription of ISGs to confront BoHV-1 replication in MDBK cells. The type 1 interferon pathway was activated due to the transcription of cGAS and IRF7 triggered in response to BoHV-1 infection. Therefore, transcription of ISGs is activated in experimental groups treated with BoIFN-γ and MDBK infected BoHV-1. Interferon regulatory transcription factors, IRF1 and GBP5, were activated by BoIFN-γ stimuli rather than BoHV-1 infection (Supplementary Figure 2). The transcription level of IRF1 was significantly higher in group MgIV than in group MIV. Previous studies have shown that IRF1 is an early-target gene downstream of IFN-γ signalling and plays a crucial antiviral role in HSV-1 infection (Irving et al., 2020). GBP5 functions as an activator of NLRP3 inflammasome assembly and is important to the innate immune system and in pathogen-inhibiting inflammation (Krapp et al., 2016). Positive regulation of IRF1 and GBP5 transcription in BoHV-1 infected MDBK cells pre-treated with BoIFN-γ was more pronounced than in group MIV. The promoter of IRF1 contains a GAS element which could be activated by STAT1 homodimers induced by the binding of IFN-γ to its receptor (Feng et al., 2021). IRF1 is an activating factor in pathogen defence and contributes to the activation of innate immune responses induced by pathogen sensors such as RLRs, TLRs, or cGAS. As described by IRF1 promotes the innate immune response to viral infection and exhibits antiviral activity both in DNA and RNA virus infection (Wang et al., 2020; Feng et al., 2021). GBP5 is mainly induced by IFN-γ and is involved in innate immunity against a wide variety of microbial pathogens. GBP5 is reported as the major effector of the anti-RSV activity of IFN-γ treatment and potently restricts HIV-1 and other retroviruses (Krapp et al., 2016; Li et al., 2020). Considering the above evidence, we speculate that the activation of IRF1 and GBP5 may play an important role in BoIFN-γ inhibition of viral replication and cytopathic effect relief. Transcription of pro-inflammatory cytokines TNF-α, IL6, IL6 receptor (IL6R) and IL12 was upregulated by BoHV-1 infection, whereas transcription of these genes is almost unaffected by BoIFN-γ treatment (Supplementary Figure 2). However, these pro-inflammatory cytokines still play critical roles in the host antiviral response.

## Conclusion

In this study, we found that pre-treatment with BoIFN-γ significantly inhibits the replication and cytopathic effects of BoHV-1 in MDBK. Tanscriptome analysis revealed that BoIFN-γ upregulated ISG transcriptions and interferon regulatory transcription factors IRF1 and GBP5 associated with the host immune response against BoHV-1 infection. In addition, BoIFN-γ promotes expression of cellular components involved in complement activation and coagulation cascades response as well as antigen processing and presentation process. Furthermore, BoIFN-γ significantly alleviated the disorder of gene transcription involved in metabolism and DNA repair caused by BoHV-1 infection. Taken together, BoIFN-γ pre-treatment can improve host cell resistance to BoHV-1 infection, probably due to regulation of gene transcriptions related to cellular metabolism and innate immune response. Although further studies are needed to confirm whether these regulatory effects of BoIFN-γ directly or indirectly inhibit BoHV-1 replication, our findings provide insights into the development of prophylactic agents for prevention and control of BoHV-1 infection.

## Data availability statement

The datasets presented in this study can be found in online repositories. The names of the repository/repositories and accession number(s) can be found at: https://www.ncbi.nlm.nih.gov/sra/PRJNA849906.

## Author contributions

YL and BJ conceived and designed the experiments, analysed the data, and wrote the paper. BJ, JW, WL, JC, JX, and MC performed the experiments. WL, JC, and JX contributed reagents, materials, and analysis tools. All authors contributed to the article and approved the submitted version.

## Funding

This work was supported by a grant from National Natural Science Foundation of China (grant no. 32172818), Beijing Agricultural Forestry Academy Youth Fund (grant no. QNJJ201930), and Beijing Innovation Team of Technology Systems in the Dairy Industry (grant no. BAIC06-2022).

## Conflict of interest

The authors declare that the research was conducted in the absence of any commercial or financial relationships that could be construed as a potential conflict of interest.

## Publisher’s note

All claims expressed in this article are solely those of the authors and do not necessarily represent those of their affiliated organizations, or those of the publisher, the editors and the reviewers. Any product that may be evaluated in this article, or claim that may be made by its manufacturer, is not guaranteed or endorsed by the publisher.

## Footnotes

^1^ http://www.kegg.jp

^2^ http://www.genome.jp/kegg
