# Supplementary material for: Comparative transcriptome analysis of MDBK cells reveals that BoIFN-γ augmented host immune responses to bovine herpesvirus 1 infection
Source: Front Microbiol. 2022 Aug 9;13:973278. doi: 10.3389/fmicb.2022.973278 (PMC9396027; doi:10.3389/fmicb.2022.973278)
Supplement: Supplementary file 1 [file Data_Sheet_1.docx]

## Supplementary Figures


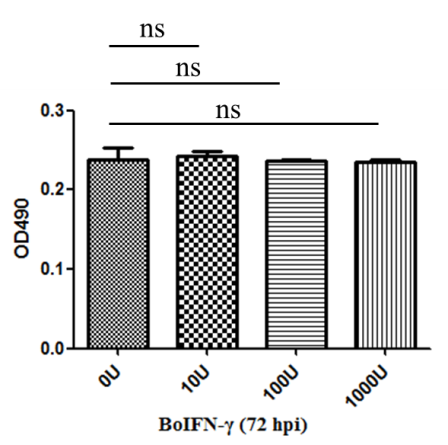


**Supplementary Figure 1 |** Cytotoxicity of BoIFN-γ to MDBK Cells

MDBK cells were seeded in 6-well plates and cultured to 70% confluency at 37 ºC for 18-24 h. Then, 1mL of BoIFN-γ with different concentrations (10 U/mL, 100 U/mL, 1000 U/mL) was added to the culture medium respectively. Followed by cultivation at 37 ºC for 12 h we change the medium contained BoIFN-γ to fresh maintenance medium and collected supernatants after 27 h. Cell proliferation was tested with the CellTiter 96 Aqueous One Solution Cell Proliferation Assay (MTS) Kit (Promega) according to the manufacturer’s instructions, and the OD490 values of the test wells were read with a microplate reader. All tests were performed in triplicate.


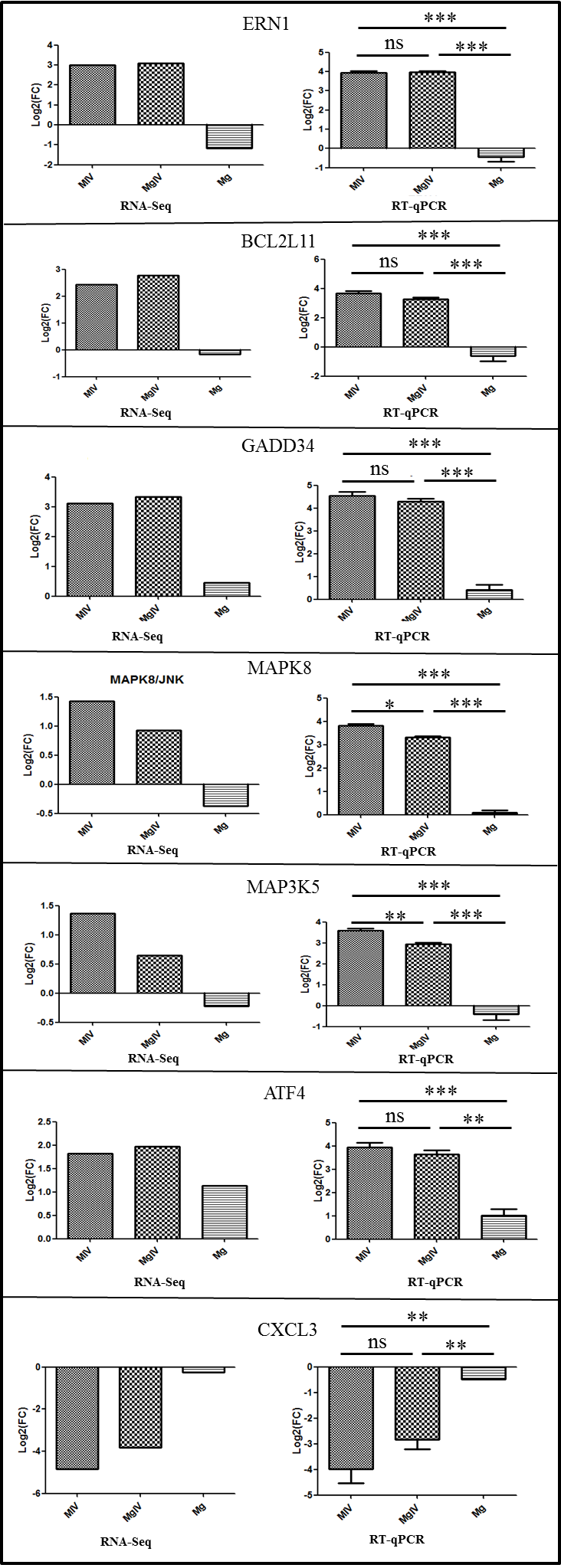


**Supplementary Figure 2 |** Expression level of ERN1, BCL2L11, GADD34, MAPK8, MAP3K5, ATF4 and CXCL3. (RNA-Seq and RT-qPCR)

## Supplementary Tables

**Supplementary Table 1 | BoIFN-γ production information**

| Source organism  DNA source | Expression vector | Expression host | Complete amino-acid sequence of the construct produced |
| --- | --- | --- | --- |
| Bos taurus  GenBank：EU276066 | pET-21a(+) | E. coli strain BL21 (DE3) | QGQFFREIENLKEYFNASSPDVAKGGPLFSEILKNWKDESDKKIIQSQIVSFYFKLFENLKDNQVIQRSMDIIKQDMFQKFLNGSSEKLEDFKKLIQIPVDDLQIQRKAINELIKVMNDLS |

**Supplementary Table 2 |** Primers for RT-qPCR in this study

| Name | Sequence (5’-3’) | Name | Sequence (5’-3’) |
| --- | --- | --- | --- |
| C3-F | TCAGAGCGGGTACCTCTTCA | C3-R | TCAGGGGTCTCGATGGTGAT |
| C1s-F | GGTGCCTGTGTTTGTTCGAC | C1s-R | TGCACCACATCTCTGGCAAT |
| ATM-F | GTGGTGGCACAAAAAGTGAAGT | ATM-R | TCGGCTTGTTTGTGAAGGGT |
| BRCA2-F | GGCCACACCACCAACACTTA | BRCA2-R | AGGCATTCAAAGCAACCACAA |
| BRCA1-F | AGGCTTCAAAGCGTCAGACA | BRCA1-R | CTGCACGTGCTGGCTTTTAG |
| BRIP1-F | GGGCTTGTTCGAAGGACAGTA | BRIP1-R | TTGTTTGCTGAAGGTTGGGC |
| ZBTB11-F | ACGCAGACGGAGAAGATTCC | ZBTB11-R | CATTTTGGAAGCGGGTAGGC |
| MAPK8-F | CCAGAATGCTGGTAATCGACG | MAPK8-R | AGGTATCTTTGGTGGTGGAGC |
| ACSS2-F | CCGGGTACTATGTGACAGGAG | ACSS2-R | CACAGGATGAGGGTGTCCAA |
| ADAR-F | TGGACCCAAGACAGGGGTAT | ADAR-R | GGAGCTGGAAACTGTCACGA |
| ERN1-F | GAACGCCACCTACTTCGACT | ERN1-R | CCACGGGGGAAGCATAGTTT |
| CXCL3-F | CACAAAGAGGACACCCCCAC | CXCL3-R | GCCGGGTGAACCCCTTTTAT |
| CD74-F | GGAGAAGCCCTTTGAGGGTC | CD74-R | GCACTGGAGCGGCATATAGT |
| ZBTB-F | ACGCAGACGGAGAAGATTCC | ZBTB-R | CATTTTGGAAGCGGGTAGGC |
| RAD51-F | TACCTTTAGGCCAGAACGGC | RAD51-R | TCCATCCCCCACAAAGCAAT |
| MX1-F | TGGACGAGTCCAAGAGCAAC | MX1-R | TGAGCTCTGGTCCCCGATAA |
| B2M-F | GGTTCCATCCACCCCAGATT | B2M-R | CGGCAGCTGTACTGATCCTT |
| BOLA-F | CGAAACTTAAAGGACGCCGC | BOLA-R | ATCCACTGGAGGGTGTGAGA |
| BOLA-DRA-F | GCAAAGCCAACACCGTTGAA | BOLA-DRA-R | TGCTGAAGCATGGTGGTCTT |
| PA28-F | CGGAGATCTAGCAGCAACTGG | PA28-R | CCTCAGCCTCCTGGAAAAGAT |
| TAPBP-F | GCGAGATGAGCCTCTACGTC | TAPBP-R | ACATGCTGACCATGAGCCAA |
| MAP3K5-F | GTGTCACTCCAGTTCTCCTGG | MAP3K5-R | TCCTCAGCCAAAAACCGACTT |
| ATF4-F | CATCATGGGTTCTCCTGCGA | ATF4-R | AGTAGTGGTGGAGTCTGGCT |
| FDFT1-F | CCAAGCCCCTATCTGGGAAA | FDFT1-R | CTCCACCACAGGTCACAGTT |
| HMGCS1-F | ACGGTGACACTAGTTCTGGAAA | HMGCS1-R | GTCAGGAGCAGACCTAGGGA |
| SQLE-F | ACTCTGGTTACTGTGGAAGCG | SQLE-R | ACTCAGCTGAACGGCACTTT |
| CLSPN-F | GACGGGTGAAGTGGTTTCCG | CLSPN-R | TGTTCACTCAAGGGCCCAAT |
| RAD54L-F | CGCATAGAACGCAGCCAATG | RAD54L-R | GTTTGGGAGGGGAATCCCAG |
| IL6-F | GCTGCTCCTGGTGATGACTT | IL6-R | GATTTTGTCGACCATGCGCT |
| IL6R-F | AGAGCCCCCTCAGTAGAGTG | IL6R-R | CCTGGAAGACTTGCTTCGGA |
| TNF-F | GGACACCCAGAATGTGAGGG | TNF-R | GGAGAGTTGAAGTCCACGCA |
| GADD34-F | AGACCAAAGACAGTGGAGCG | GADD34-R | GTTGCTTCTTCCCCACCGTA |
| BCL2L11-F | CAATGGCTTCCATGAGGCAG | BCL2L11-R | ACCAGACGCACGATGTAGC |
| EIF2AK2-F | GCTTTGGGGCTGATTCTTGC | EIF2AK2-R | GCTTTGGGGCTGATTCTTGC |
| β-actin-F | GATATTGCTGCGCTCGTGGT | β-actin-R | CATCCCCCACGTACGAGTC |

*F represents forward PCR primer; R represents reverse PCR primer.

**Supplementary Table 3 |** The antiviral activity of BoIFN-γ

| Dilution of BoIFN-γ | Number of walls Incubated | Number of  walls with CPE | Number of walls without CPE | Totle Number | | | Protection efficiency  （%） |
| --- | --- | --- | --- | --- | --- | --- | --- |
|  |  |  |  | Number of walls with CPE | Number of walls without CPE | Number of walls |  |
| 10^-2^ | 8 | 0 | 8 | 0 | 24 | 24 | 100.00 |
| 10^-3^ | 8 | 1 | 7 | 1 | 16 | 17 | 94.12 |
| 10^-4^ | 8 | 3 | 5 | 4 | 9 | 13 | 69.23 |
| 10^-5^ | 8 | 5 | 3 | 9 | 4 | 13 | 30.77 |
| 10^-6^ | 8 | 7 | 1 | 16 | 1 | 17 | 5.88 |
| 10^-7^ | 8 | 8 | 0 | 24 | 0 | 24 | 0 |
| 10^-8^ | 8 | 8 | 0 | 32 | 0 | 32 | 0 |
| 10^-9^ | 8 | 8 | 0 | 40 | 0 | 40 | 0 |
| Negative control | 8 | 0 | 8 | 0 | 8 | 8 | 0 |
| Positive control | 8 | 8 | 0 | 8 | 0 | 8 | 0 |
| Blank control | 8 | 0 | 8 | 0 | 8 | 8 | 0 |

Distance ratio=(69.23-50)/(69.23-30.77)=0.5, the anti-BoHV-1 activity of the BoIFN-γ in MDBK=10^4.5^U/0.1ml=3.16×10^5^U/ml，and as the concentration of BoIFN-γ is 0.668mg/ml, the anti-BoHV-1 activity of the BoIFN-γ in MDBK is 4.73×10^5^U/mg.

**Supplementary Table 4 |** KEGG pathway classification of differently expressed genes (MIV vs Mock)

| Description | Pathway ID | DEGs | P-Value |
| --- | --- | --- | --- |
| Oxidative phosphorylation | bta00190 | 91 | 1.60E-06 |
| Ribosome | bta03010 | 98 | 4.31E-06 |
| Parkinson disease | bta05012 | 93 | 5.19E-06 |
| Metabolic pathways | bta01100 | 605 | 1.99E-05 |
| Alzheimer disease | bta05010 | 100 | 6.49E-05 |
| Herpes simplex virus 1 infection | bta05168 | 193 | 6.64E-05 |
| Huntington disease | bta05016 | 105 | 0.000167 |
| Thermogenesis | bta04714 | 120 | 0.000324 |
| Non-alcoholic fatty liver disease (NAFLD) | bta04932 | 86 | 0.00033 |
| Lysosome | bta04142 | 64 | 0.003889 |
| Ribosome biogenesis in eukaryotes | bta03008 | 45 | 0.008132 |
| Propanoate metabolism | bta00640 | 22 | 0.011598 |
| Carbon metabolism | bta01200 | 56 | 0.013381 |
| Glyoxylate and dicarboxylate metabolism | bta00630 | 20 | 0.015664 |
| Phagosome | bta04145 | 78 | 0.016552 |
| Taste transduction | bta04742 | 41 | 0.018461 |
| Cysteine and methionine metabolism | bta00270 | 28 | 0.020225 |
| Cellular senescence | bta04218 | 73 | 0.037629 |
| Fatty acid metabolism | bta01212 | 30 | 0.040513 |
| Cell cycle | bta04110 | 56 | 0.040905 |
| Valine, leucine and isoleucine degradation | bta00280 | 27 | 0.041718 |
| Toxoplasmosis | bta05145 | 52 | 0.041802 |
| Retrograde endocannabinoid signaling | bta04723 | 67 | 0.043314 |
| Human T-cell leukemia virus 1 infection | bta05166 | 98 | 0.044353 |

**Supplementary Table 5 |** KEGG pathway classification of differently expressed genes (MgIV vs Mock)

| Description | Pathway ID | DEGs | P-Value |
| --- | --- | --- | --- |
| Glyoxylate and dicarboxylate metabolism | bta00630 | 15 | 0.000629 |
| Lysosome | bta04142 | 37 | 0.000799 |
| MAPK signaling pathway | bta04010 | 70 | 0.001576 |
| Valine, leucine and isoleucine degradation | bta00280 | 19 | 0.002148 |
| Fatty acid metabolism | bta01212 | 20 | 0.003451 |
| Carbon metabolism | bta01200 | 31 | 0.006298 |
| beta-Alanine metabolism | bta00410 | 13 | 0.01241 |
| FoxO signaling pathway | bta04068 | 33 | 0.014983 |
| Metabolic pathways | bta01100 | 268 | 0.015958 |
| Glutathione metabolism | bta00480 | 18 | 0.016396 |
| Human T-cell leukemia virus 1 infection | bta05166 | 52 | 0.018557 |
| Biosynthesis of unsaturated fatty acids | bta01040 | 11 | 0.018951 |
| Arginine and proline metabolism | bta00330 | 15 | 0.020575 |
| Histidine metabolism | bta00340 | 9 | 0.02404 |
| Toxoplasmosis | bta05145 | 28 | 0.025639 |
| Propanoate metabolism | bta00640 | 11 | 0.031362 |
| Fatty acid elongation | bta00062 | 10 | 0.033282 |
| Melanoma | bta05218 | 19 | 0.041141 |
| Biosynthesis of amino acids | bta01230 | 19 | 0.041141 |
| Ribosome biogenesis in eukaryotes | bta03008 | 21 | 0.041162 |
| Ubiquinone and other terpenoid-quinone biosynthesis | bta00130 | 5 | 0.043595 |
| Other glycan degradation | bta00511 | 8 | 0.043771 |
| Gastric cancer | bta05226 | 34 | 0.048729 |
| Central carbon metabolism in cancer | bta05230 | 17 | 0.049755 |

**Supplementary Table 6 |** KEGG pathway classification of differently expressed genes (Mg vs Mock)

| Description | Pathway ID | DEGs | P-Value |
| --- | --- | --- | --- |
| Antigen processing and presentation | bta04612 | 34 | 8.14E-10 |
| Allograft rejection | bta05330 | 27 | 1.91E-09 |
| Graft-versus-host disease | bta05332 | 25 | 2.25E-09 |
| Viral myocarditis | bta05416 | 30 | 1.00E-08 |
| Type I diabetes mellitus | bta04940 | 26 | 1.62E-08 |
| Epstein-Barr virus infection | bta05169 | 55 | 4.56E-08 |
| Influenza A | bta05164 | 46 | 1.04E-07 |
| Staphylococcus aureus infection | bta05150 | 27 | 2.43E-07 |
| Autoimmune thyroid disease | bta05320 | 26 | 3.08E-07 |
| Inflammatory bowel disease (IBD) | bta05321 | 25 | 6.07E-07 |
| Leishmaniasis | bta05140 | 26 | 8.90E-07 |
| Phagosome | bta04145 | 41 | 2.22E-06 |
| Herpes simplex virus 1 infection | bta05168 | 74 | 3.77E-06 |
| Asthma | bta05310 | 17 | 4.09E-06 |
| Intestinal immune network for IgA production | bta04672 | 20 | 9.35E-06 |
| Human T-cell leukemia virus 1 infection | bta05166 | 48 | 1.47E-05 |
| Cell adhesion molecules (CAMs) | bta04514 | 36 | 2.87E-05 |
| Toxoplasmosis | bta05145 | 28 | 5.66E-05 |
| Th1 and Th2 cell differentiation | bta04658 | 24 | 0.000216 |
| Rheumatoid arthritis | bta05323 | 24 | 0.000357 |
| Th17 cell differentiation | bta04659 | 25 | 0.000596 |
| Tuberculosis | bta05152 | 37 | 0.000634 |
| Pertussis | bta05133 | 19 | 0.000867 |
| Hematopoietic cell lineage | bta04640 | 24 | 0.000897 |
| Complement and coagulation cascades | bta04610 | 20 | 0.001354 |
| p53 signaling pathway | bta04115 | 17 | 0.004263 |
| NOD-like receptor signaling pathway | bta04621 | 30 | 0.00876 |
| Glutathione metabolism | bta00480 | 12 | 0.027155 |
| Measles | bta05162 | 24 | 0.027657 |
| Endocytosis | bta04144 | 35 | 0.032762 |
| Biosynthesis of amino acids | bta01230 | 13 | 0.044788 |

**Supplementary Table 7 |** KEGG pathway classification of differently expressed genes (MgIV vs MIV)

| Description | Pathway ID | DEGs | P-Value |
| --- | --- | --- | --- |
| Staphylococcus aureus infection | bta05150 | 5 | 4.77E-06 |
| Leishmaniasis | bta05140 | 4 | 0.000108 |
| Antigen processing and presentation | bta04612 | 4 | 0.000163 |
| Phagosome | bta04145 | 5 | 0.000204 |
| Asthma | bta05310 | 3 | 0.000269 |
| Systemic lupus erythematosus | bta05322 | 5 | 0.000277 |
| Tuberculosis | bta05152 | 5 | 0.000403 |
| Graft-versus-host disease | bta05332 | 3 | 0.000514 |
| Intestinal immune network for IgA production | bta04672 | 3 | 0.000789 |
| Allograft rejection | bta05330 | 3 | 0.000789 |
| Type I diabetes mellitus | bta04940 | 3 | 0.000912 |
| Inflammatory bowel disease (IBD) | bta05321 | 3 | 0.001406 |
| Autoimmune thyroid disease | bta05320 | 3 | 0.001522 |
| Viral myocarditis | bta05416 | 3 | 0.001836 |
| Th1 and Th2 cell differentiation | bta04658 | 3 | 0.003688 |
| Rheumatoid arthritis | bta05323 | 3 | 0.004112 |
| Hematopoietic cell lineage | bta04640 | 3 | 0.005047 |
| Th17 cell differentiation | bta04659 | 3 | 0.005427 |
| Toxoplasmosis | bta05145 | 3 | 0.005427 |
| Herpes simplex virus 1 infection | bta05168 | 5 | 0.008495 |
| Cell adhesion molecules (CAMs) | bta04514 | 3 | 0.013472 |
| Influenza A | bta05164 | 3 | 0.018066 |
| Pertussis | bta05133 | 2 | 0.024337 |
| Complement and coagulation cascades | bta04610 | 2 | 0.030334 |
| Epstein-Barr virus infection | bta05169 | 3 | 0.033833 |
| Human T-cell leukemia virus 1 infection | bta05166 | 3 | 0.036082 |

**Supplementary Table 8 |** KEGG pathway classification of differently expressed genes (MgIV vs Mg)

| Description | Pathway ID | DEGs | P-Value |
| --- | --- | --- | --- |
| Antigen processing and presentation | bta04612 | 38 | 3.51E-05 |
| Graft-versus-host disease | bta05332 | 25 | 0.00012 |
| Lysosome | bta04142 | 45 | 0.000311 |
| Valine, leucine and isoleucine degradation | bta00280 | 24 | 0.000515 |
| Phagosome | bta04145 | 56 | 0.00055 |
| Metabolic pathways | bta01100 | 344 | 0.000572 |
| Epstein-Barr virus infection | bta05169 | 70 | 0.000638 |
| Type I diabetes mellitus | bta04940 | 26 | 0.00067 |
| Human T-cell leukemia virus 1 infection | bta05166 | 71 | 0.000764 |
| Toxoplasmosis | bta05145 | 39 | 0.001796 |
| Leishmaniasis | bta05140 | 29 | 0.002037 |
| Viral myocarditis | bta05416 | 29 | 0.002037 |
| Inflammatory bowel disease (IBD) | bta05321 | 27 | 0.002148 |
| Arginine and proline metabolism | bta00330 | 21 | 0.002263 |
| Allograft rejection | bta05330 | 23 | 0.002729 |
| Pertussis | bta05133 | 28 | 0.004154 |
| Intestinal immune network for IgA production | bta04672 | 22 | 0.005077 |
| Rheumatoid arthritis | bta05323 | 34 | 0.005249 |
| Staphylococcus aureus infection | bta05150 | 27 | 0.00537 |
| Glyoxylate and dicarboxylate metabolism | bta00630 | 14 | 0.007563 |
| Th1 and Th2 cell differentiation | bta04658 | 32 | 0.008402 |
| Carbon metabolism | bta01200 | 35 | 0.011499 |
| Th17 cell differentiation | bta04659 | 35 | 0.011499 |
| Fatty acid metabolism | bta01212 | 21 | 0.012398 |
| Biosynthesis of unsaturated fatty acids | bta01040 | 13 | 0.015484 |
| Asthma | bta05310 | 15 | 0.01811 |
| Hematopoietic cell lineage | bta04640 | 33 | 0.019585 |
| beta-Alanine metabolism | bta00410 | 14 | 0.024062 |
| Autoimmune thyroid disease | bta05320 | 23 | 0.024416 |
| Propanoate metabolism | bta00640 | 13 | 0.027014 |
| Steroid biosynthesis | bta00100 | 9 | 0.027704 |
| Biosynthesis of amino acids | bta01230 | 23 | 0.030722 |
| Sulfur metabolism | bta00920 | 6 | 0.032923 |
| Ribosome biogenesis in eukaryotes | bta03008 | 25 | 0.036953 |
| p53 signaling pathway | bta04115 | 23 | 0.046912 |
